# Integrating Patient-Generated Health Data Into Clinical Care Settings or Clinical Decision-Making: Lessons Learned From Project HealthDesign

**DOI:** 10.2196/humanfactors.5919

**Published:** 2016-10-19

**Authors:** Deborah J Cohen, Sara R Keller, Gillian R Hayes, David A Dorr, Joan S Ash, Dean F Sittig

**Affiliations:** ^1^ Department of Family Medicine Oregon Health & Science University Portland, OR United States; ^2^ Donald Bren School of Information and Computer Sciences Department of Informatics University of California, Irvine Irvine, CA United States; ^3^ Department of Medical Informatics and Clinical Epidemiology Oregon Health & Science University Portland, OR United States; ^4^ Memorial Hermann Center for Healthcare Quality & Safety School of Biomedical Informatics University of Texas Houston, TX United States

**Keywords:** mobile applications, chronic disease, self-management, doctor-patient relations

## Abstract

**Background:**

Patient-generated health data (PGHD) are health-related data created or recorded by patients to inform their self-care and understanding about their own health. PGHD is different from other patient-reported outcome data because the collection of data is patient-driven, not practice- or research-driven. Technical applications for assisting patients to collect PGHD supports self-management activities such as healthy eating and exercise and can be important for preventing and managing disease. Technological innovations (eg, activity trackers) are making it more common for people to collect PGHD, but little is known about how PGHD might be used in outpatient clinics.

**Objective:**

The objective of our study was to examine the experiences of health care professionals who use PGHD in outpatient clinics.

**Methods:**

We conducted an evaluation of Project HealthDesign Round 2 to synthesize findings from 5 studies funded to test tools designed to help patients collect PGHD and share these data with members of their health care team. We conducted semistructured interviews with 13 Project HealthDesign study team members and 12 health care professionals that participated in these studies. We used an immersion-crystallization approach to analyze data. Our findings provide important information related to health care professionals’ attitudes toward and experiences with using PGHD in a clinical setting.

**Results:**

Health care professionals identified 3 main benefits of PGHD accessibility in clinical settings: (1) deeper insight into a patient’s condition; (2) more accurate patient information, particularly when of clinical relevance; and (3) insight into a patient’s health between clinic visits, enabling revision of care plans for improved health goal achievement, while avoiding unnecessary clinic visits. Study participants also identified 3 areas of consideration when implementing collection and use of PGHD data in clinics: (1) developing practice workflows and protocols related to PGHD collection and use; (2) data storage, accessibility at the point of care, and privacy concerns; and (3) ease of using PGHD data.

**Conclusions:**

PGHD provides value to both patients and health care professionals. However, more research is needed to understand the benefit of using PGHD in clinical care and to identify the strategies and clinic workflow needs for optimizing these tools.

## Introduction

Patients with chronic conditions often require continuous management of care rather than brief, single-focused interventions [1]. Data collected between visits can inform ongoing care management and provide important insights into a patient’s health and well-being. As technology advances and patients also become more actively engaged in producing their own health data, the amount of health data produced grows substantially [2].

Patient-generated health data (PGHD) are “data created, recorded, and gathered by and from patients” [3]. Generating and capturing PGHD (eg, physical activity, food consumption) are increasingly common, particularly among patients with chronic illnesses [4-14]. Observations of daily living (ODLs) are one type of PGHD and are defined as patient observations and recordings of “the patterns and realities of daily life...diet, physical activity, quality and quantity of sleep, pain episodes, and mood” [15]. ODLs are unique from other types of PGHD because they are patient-informed; patients record the aspects of daily life they identify as most relevant to track [16]. These decisions can be made individually or in collaboration with health care professionals, and ODLs such as PGHD can be used for personal tracking and improvement as well as to inform clinical care [13,17,18]. PGHD collected through a range of different types of smart devices and other new technologies can provide patients with innovative ways to actively manage their health [11,19-21], improving patient self-knowledge [12], and management of health concerns, including diabetes [4-6,14,22], physical activity [8,10], and behavioral health triggers such as anxiety [7,9,23].

PGHD is distinguished from other types of patient experience data, such as patient-reported outcomes (PRO) (eg, NIH PROMIS) [24] and data generated through ecological momentary assessment (EMA) [25,26]. PRO data are standardized questions and surveys designed to understand patients’ experiences of health such as mood and work-life function. The collection of PRO data are stimulated, driven, and informed by health care professionals and practices, and data are often collected through clinic-based tools such as electronic health records (EHRs) or patient portals [27-29]. In contrast, PGHD is patient-directed and patient-informed and is not collected through clinic tools but through a range of readily available commercial off-the-shelf tools such as mobile phone apps and wearable activity trackers [22]. PGHD is also distinguished from EMA, which is a data collection method where study participants repeatedly report on, for instance, a symptom or behavior [19]. While reporting is done in the natural environment, as is the case with PRO, what is measured is researcher-driven, not patient-driven. Additionally, the purpose of collecting EMA data is research, not self-management of one’s health, as is the case with PGHD.

PGHD are a unique and important type of data relevant for health care settings, and for informatics experts that support information management in these settings. PGHD has the potential to impact health care delivery, and the patient-clinician relationship [21,30-32], including the possibility of reducing the demand for face-to-face clinic visits [33,34]. However, little is known about health care professionals’ experiences with PGHD and their willingness to use it in the outpatient setting [33,35,36]. We begin to fill this gap by examining the experiences of health care professionals, working in a range of outpatient settings, who cared for patients collecting PGHD.

## Methods

### Setting

We conducted an evaluation of Project HealthDesign (PHD), a US national program of the Robert Wood Johnson Foundation that involved 2 rounds of funding. This paper focuses on Round 2 of the PHD program conducted between April 2010 and July 2012, which required grantees to develop and pilot innovative health information technology (health IT) tools to enable PGHD collection with the goal of using that information to promote patient engagement and to inform both personal health management and clinical decision-making. The program funded 5 US-based academic research teams who worked with patients to support the collection of PGHD through a range of apps, sensors, and/or websites.

### Sample

The sample for this study constitutes the 5 studies funded through the PHD program, which provided a unique opportunity to identify a group of health care professionals that had been exposed to using a range of different PGHD in clinical care. We invited health care professionals, including clinicians, nurses, and health coaches associated with each of the 5 PHD studies for interviews. We interviewed 12 health care professionals who cared for patients using health IT tools. In addition, we invited study team members who worked closely with health care professionals to participate in interviews because they had important experiences and perspectives on health care professionals use of these data in the clinic. We interviewed 13 PHD study team members participating in the PHD studies to understand, from their perspective, the benefits and challenges health care professionals experienced when using PGHD. This study was approved by the Institutional Review Board of Oregon Health & Science University.

### Data Collection

As the evaluators of the PHD program, we had access to documents about each PHD study, including details about their project (eg, aims, study design, study team, participants), the tools they developed, and access to some data they collected as part of their own evaluations. We used this to gain an understanding of each PHD study and identify interview participants.

DJC and SRK conducted one-on-one, semistructured interviews with participants, either in person or by Internet-enabled video conferencing software (eg, Skype, Microsoft Inc., Redmond, WA, USA). We had to conduct 3 interviews by telephone, but we did not have access to visually-based nonverbal behaviors.

Our team developed 2 interview guides, one for health care professionals and the other for study team members. Both these guides were quite similar and focused on asking about the background of the individuals, their roles in the practices or the study, their understandings or definitions of PGHD, their experiences using PGHD with patients (health care professionals) or experiences helping health care professional use these data (study team members), and what they thought about how PGHD might be integrated into routine clinical care processes.

Data collection and analysis was an iterative process, wherein we conducted one or two interviews, analyzed them, and used emerging findings to refine the interview guides as needed, and monitored when saturation was reached. Saturation is the point at which themes repeat during the data collection process and no new findings emerge. In our study, we hit saturation after completing the interviews of 10 health care professionals and 11 study team members, after which we conducted 4 additional interviews to confirm or disconfirm preliminary findings.

### Interview Data Management

Interviews were audio-recorded and professionally transcribed. We de-identified transcripts and entered them into Atlas.ti (version 7.0, Atlas.ti Scientific Software Development GmbH, Berlin, Germany) for data management and analysis. Study data were saved on a password-protected networked drive maintained by Oregon Health & Science University.

### Analysis

We used an immersion-crystallization approach [37] to analyze data, meaning that we approached data without *a priori* hypotheses in mind but rather with the aim of identifying emergent findings. Due to the uniqueness of each PHD study, we engaged in this process for each case (a single PHD study) first. We started by analyzing the documents and information we had about each PHD study to identify the study’s focus (eg, patients with asthma), the study’s purpose (what kind of tool was developed and what the team was testing), and who, if anyone, in the clinical setting was exposed to PGHD. Next, we analyzed interview data to examine health care professionals’ experiences when exposed to PGHD. We examined how they used these data with patients, explored how clinical processes accommodated the use of these data, and what concerns and benefits they saw for future use of these data in clinical care. We paid particular attention to similarities and differences in viewpoints among health care professionals and study team members.

After the single case analyses were completed, we conducted a cross-case comparison to identify patterns and variations across PHD studies. During this phase, we paid particular attention to variations in how PGHD were integrated into clinical practices based on the type of disease or illness and care setting, and to other factors influencing use of these data at the point of care. In the final phase, we iterated the preliminary results with consultants DAD, DFS, JSA, and GRH for feedback and to gain a multi-disciplinary perspective on the data and our preliminary findings. We used this input to refine our findings.

We further specified observed variation to the extent in which PGHD were integrated into clinical care, as part of the refinement process. We developed the following rating system: Highly integrated – PGHD were integrated clinical workflows and protocols of the doctor-nurse dyads; moderately integrated – there were no formal workflow changes to accommodate PGHD use, but some health care professionals reviewed data with patients, but this was outside of the typical care process; and minimally integrated – it was left to patients whether or not to share data with health care professionals; use of PGHD in the context of a clinical visit was minimal.

## Results

### PHD Study Attributes

Participants in the 5 PHD studies we examined included adults living with asthma (Project 1), elders at risk for cognitive decline (Project 2), overweight young adults (Project 3), people living with Crohn disease (Project 4), and caregivers of premature infants (Project 5). The involvement of the health care team (ie, how the care team received and acted on data), the focus of each project, the tools that were developed for collecting PGHD, and the extent to which PGHD were integrated into primary clinical workflows varied, as described in Table 1. In what follows, we triangulated data from health care professionals and study team members to understand the value of PGHD in clinical care, the experiences implementing PGHD into the clinical care setting, and features that support integration of PGHD data into clinical processes.

**Table 1:** Description of PHD studies and level of integration.

Study ID	Project overview	Data patients collected (PGHD)^a^	Type of health care professionals that used PGHD	Extent to which PGHD integrated into care	How PGHD were integrated into clinical workflow
1	Primary health issue: Moderate to severe asthma Patients used an app to collect health-related PGHD. Health data from these PGHDs were monitored by nurses at their primary care clinic	Medication usage, environmental factors, peak flow measurements	Primary care physician or nurse dyads	High	Nurses review data weekly via a Web-based dashboard for high-risk patients Nurses reported to their team clinician for patients who were at highest risk Nurses followed a standard protocol for interacting with the patient (phone call to change treatment, scheduling patient)
2	Primary health issue: Elders at risk for cognitive decline Passive sensors, connected to a remote server, were placed in elders’ homes to collect PGHD data. Elders and caregivers could use summary data to identify likelihood of decline	Assessed task completion (making coffee) as proxy for cognitive decline (ability to correctly sequence tasks)	Health care professionals were not exposed to PGHD in clinical settings	Minimal	Data were not integrated into clinical care Patients had the option to decide how, when, or if they shared summaries with care providers
3	Primary health issue: Adolescent behavioral health Participants (from local high school and hospital) tracked a variety of ODLs^b^ on an app. Participants also met regularly with a health coach to review ODL data and set goals	Food intake, physical activity, mood	Health coaches employed in primary care practice	Moderate	Health coach reviewed PGHD data on a Web-based dashboard and used data to support patient behavior change Health coach monitored for mental health “red flags” and reported them to appropriate health care provider
4	Primary health issue: Crohn disease Patient participants met with a physician to develop a list of ODLs to be tracked	Weight, physical activity, mood, and symptoms relevant to their illness	Gastroenterologists	Moderate	Patients had the option to share data with physician during regular visits Health care professionals reviewed data as part of visit and this informed treatment decisions
5	Primary health issue: Problems associated with premature infants Case manager of a high-risk infant follow-up program worked with caregivers of high-risk infants and reviewed PGHD	Infant’s weight, food consumption, elimination patterns	High-risk infant case managers	Moderate	Case manager reviewed data on Web-based platform (daily) Interactions with caregivers via appointment reminders and messages Caregiver’s discretion to share data with other providers

^a^ODLs: observations of daily living.

^b^PGHD: patient-generated health data.

### Health Care-Related Perspectives on PGHD

Health care professionals and PHD study team members reported that PGHD fostered a deeper and more accurate understanding of a patient’s illness through tracking of key symptoms and reported having better informed visits with patients who collected PGHD. This is because PGHD helped clinicians identify and understand how patients’ symptoms varied over longer periods of time, helped them to catch problems that might otherwise go unnoticed, and helped them and their patients better manage their disease:

The weight data turned out to be a great proxy for someone's health status with Crohn's because it actually fluctuates significantly week-to-week. And, you know, it might be sometimes like ten pounds. I mean it's amazing the things you can catch on a Wi-Fi scale if you step on it every day. And the providers were really enthusiastic about that data, especially because they only see weigh-ins like every three months or so. And so this allowed, I think, patients and providers to have a really clear starting point into sort of the ebb and flow of how things were actually progressing.

Project 4, Study Team Member

(PGHD gives me) more of an appreciation and more empathy for what patients go through on a day-to-day basis. Because it's so easy for us when we see the patient in the clinic. Like, oh, tell me about your bowel frequency over the last few days or last week. And they give a range or give you a number and I'm like, okay good, I'm glad that you're doing well. But when you're forced to look at all this additional information in the way of like, oh, well, actually this is just a good week. For the past month prior you were actually doing really badly.

Project 4, Nurse Practitioner

Health care professionals also reported that the ability to monitor and assess patient data between clinic visits allowed them to identify patients who were not reaching health care goals. For example, in Project 1, nurses monitored asthma patients’ peak flow readings and reported contacting patients by phone to refine treatments or reeducate patients about medication use. As one clinician in Project 3 noted, there were benefits to keeping patients out of the clinic by managing their treatments at home: “The more we can keep people home and giving us the information that we need to know...the better.”

### Practice Protocols for PGHD Collection and Use

Health care professionals recognized that if PGHD were to become a routine part of practice, they might have some responsibility in engaging patients in collecting PGHD. A clinician participating in Project 1 speculated that patient engagement might be a 2-step process in his practice involving assessment of the patient’s level of asthma control, then engagement of those patients who would benefit from using the asthma application:

I could envision a two-step process where you would make that part of your standard asthma visit. You would either do the asthma control test or you would do a consistent defined asthma controlled assessment. Those (patients) that are truly intermittent or mild and well-controlled maybe don’t really need this. But the folks that are scoring as uncontrolled and/or the folks that thought they were well controlled, but when you actually do the assessment they're really not, those might be the folks who this would be a more high yield tool for integrating.

Project 1, Clinician

In addition, health care professionals reported that there might be a need to negotiate with patients when determining which data elements to collect:

If a patient had chronic inflammation or if they had perianal disease or they had small bowel disease, then I would set up generic template based on what the disease location is. These are the items I think might be helpful for us to monitor going forward. And then I'd leave it open-ended if there was anything else that they want to gather, so that there's some buy-in to more than just what I want to collect. (Chuckles) And then I would go from there, and I would just have like a generic, kind of prescription so to speak, on each patient depending on where their disease is.

Project 4, Nurse Practitioner

In addition, health care professionals reported that they would need to set patients’ expectations for communication about PGHD, letting patients know that they would not be contacted if everything was normal.

When the staff was responsible for reviewing and acting on PGHD, both health care professionals and study staff reported that practice protocols were needed to guide staff decisions and actions. For example, in Project 1, patients collected peak flow measurements, asthma triggers, and medication usage. Using these data, the Web-based dashboard assigned patients to one of three zones of asthma control: Poor control (indicated by a red flag), moderate control (indicated by a yellow flag), and good control (indicated by a green flag). Each physician’s nurse reviewed the dashboard weekly, and one nurse describes how it guided her actions:

I know definitively if they were in the red zone all the time, I would call them. Sometimes I'd even just make sure they were using their medications correctly, because sometimes you'd see where they were using rescue meds, but they weren't using their controller medications. So, I would look at it and look for the red flags, so to speak, the little things that were red that said that they were having problems, or where their peak flow wasn't where we wanted it to be

Project 1, Asthma, Nurse

**Figure 1:**
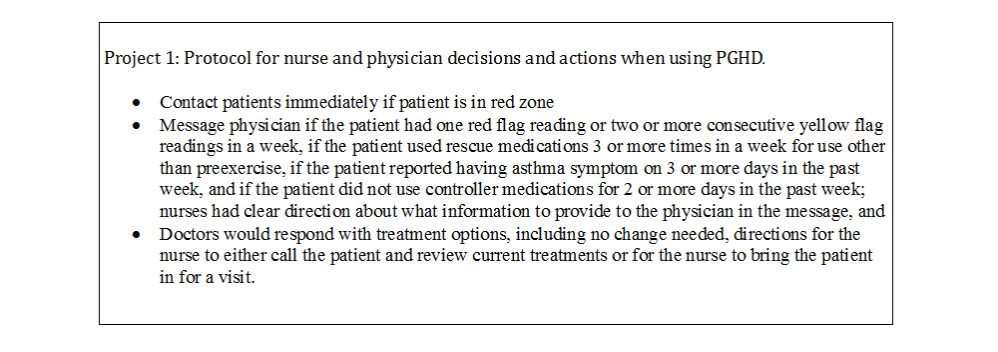
An example of practice protocol for using patient-generated health data (PGHD).

This nurse knew definitively when to contact patients in the red zone because the practice had developed a protocol to guide this behavior (see Figure 1).

### Data Storage, Accessibility, and Privacy, and Ease of Using PGHD

In addition to the practice changes identified above, health care professionals and PHD study teams identified data storage, accessibility at the point of care, and privacy concerns, and ease of using PGHD as important areas to consider when implementing health IT tools to assist patients with collecting PGHD.

#### Data Storage, Accessibility, and Privacy

PHD study teams handled data storage for participating clinical practices and avoided integrating the data patients’ generated into practices’ EHRs. Using a range of applications, data were protected on study team servers, and made available to health care professionals through Web-based platforms. Health care professionals reported wanting data integrated in the EHR to make it easier to use and more accessible at the point of care. For example, one nurse described her experience:

What we had to do was go into another window, you know, go into... I went into it. Then I had to type in my password again. But it would be nice if it were actually something that could be part of the EHR where you just click on it and it pops up.

Project 1, Nurse

Health care professionals’ preference to have PGHD integrated in the EHR was tempered by legal concerns related to patient privacy and data storage. Health care professionals and study team members reported that, outside of the study context, data storage would be the responsibility of clinical practices, the larger health care organization, or a third party who would host these data; they questioned whether “third party” data storage providers would be Health Insurance Portability and Accountability Act (HIPAA)-compliant.

Health care professionals also reported concerns about security of communications with patients and maintenance of privacy and confidentiality. For example, in the study in which health coaches were using standard mobile phone texting functionality to communicate with adolescents about their health behaviors, they shared concerns that someone other than the teen (eg, a parent) might see these communications:

There are some things that when they talk to us about sexually related issues, substance abuse, mental health, after age 12, they’re protected from us talking to their parents about it. There would be a selective bias, you know, probably about what they enter. So if they’re drunk or had a wild weekend and had some sexual partners or something, I’m not going to put that in here.

Project 3, Health Coach

Out of concern that parents might view the messages, health care professionals reported taking precaution when determining what they would communicate to adolescents via text.

#### Ease of Use: Synthesizing and Visualizing Tools for PGHD

All 5 PHD projects provided health care professionals with data dashboards, which are Web-based tools that aggregate, summarize, and visualize PGHD. Participants reported that the ability to sort or summarize data in a descriptive manner, or to graph it in different ways, helped health care professionals to more quickly see patterns in the data patients’ generated, and to extrapolate something meaningful from these data:

Because that was our concern from a provider standpoint that just going through this much data was going to be so time consuming. So that ability to put all of the data on top of each other, transpose it so we could see all the graphs at once, and see if anything correlated was helpful.

Project 4, Nurse Practitioner

In addition to being able to visualize and manipulate data, health care professionals reported the need to customize their dashboards. For example, the nurse care manager in charge of reviewing the data for Project 5 reported that she customized her dashboard homepage so the most relevant patient information (in this case, alerts) was the easiest to see. As the following example from the asthma project shows, if the information displayed in a Web-based dashboard could not easily be rearranged to meet user needs, it reduced ease of use and efficiency:

(Referencing the dashboard) ...you pulled up the list of everybody. And then you were like, okay, now where are my patients? They weren't necessarily even in alphabetical order. When they added them on, they might have been on the second page. But now because we have more, they bumped them to another page. Yeah. So that would be great if you had folders that you could just say, these are the patients I'm following and put them in that folder. And you could open your folder and all your patients would be right there. That would be good.

Project 1, Nurse

In the above quote, the screen described by the nurse displayed patient information for all the patients in the study, yet nurses only needed to locate and see data on their own team’s patients. The trouble, the nurse notes, is the inability to sort the data by clinician, and this added time to her tasks.

## Discussion

### Principal Findings

The data patients collect about their own health-related activities, support their self-management activities [19,30,38], and may also inform their interactions with health care professionals [30]. Our study of the Round 2 PHD projects shows that health care professionals recognize both the potential value of using PGHD in the clinical care process and the potential concerns that may arise related to data storage, privacy, and clinic workflow. We highlight some of these issues that must be addressed when making PGHD part of the formal clinical care process. Our work contributes to a nascent body of research identifying what motivates patients to collect data about themselves [39] and also how these data might be useful in clinical care [20,22,40-43].

We found that the usefulness of PGHD in the outpatient setting rests not only on data having clinical relevance but also relies on patients to collect these data. There is recognition among health care professions that using PGHD tools to inform health care requires balancing the data patients want to and are willing to collect with the data health care professionals find valuable. While little has been written about how to strike this balance in the PGHD development process, most of the PHD teams engaged multiple users in the development of tracking tools. Those interested in developing PGHD tools might benefit from reviewing published work in the area of groupware development, which identifies steps for developing products to benefit different types of group members [44].

Requirements must be met to use PGHD in clinical outpatient settings. For example, PGHD needs to be summarized so that patterns can be easily visualized by health care professionals who also saw benefits in being able to manipulate these data. Both of these functions were important for rapid sensemaking and decision-making [2,36,45]. The effort and cost that goes into making PGHD data useful at the point of care not only adds value but may influence whether or not health professionals utilize these tools and/or the data generated by their patients. Operational issues to consider include ensuring secure data storage and developing standards and guidelines for patient privacy [45,46], which may include teaching patients the basics of protecting their own data [47], as providing hardware and software to support patients in collecting these data, as well as clinic team members available to train patients in tracking data. These needs, which span health IT functionality and practice operations, need to be fully considered when integrating the collection and use of PGHD into clinical settings [2].

Health care professionals reported a preference for integrating PGHD into practice-operating structures and existing clinical infrastructure, such as the EHR. However, other options, such as keeping these data as part of a patient-owned record, may be more viable and avoid some privacy concerns also identified by health care professionals. Importantly, such an approach would keep ownership of these data with the patients generating it and leave it to their discretion how, when, and/or if these data are shared with health care professionals. Patient ownership is an important characteristic of PGHD, one that distinguishes it from other types of patient experience data, such as PRO data. Keeping ownership of these data with the patients changes the position of health care professionals (to one where they are negotiating a “prescription” for data collection with patients), and this represents a level of patient empowerment and autonomy that is not always present in clinician-driven health care. In such an empowered relationship, patients might begin to expect a different level of service and engagement than is typical in the current landscape. In addition, keeping ownership of these data with patients might avoid some of the legal complications of having the outpatient organization maintain PGHD. (For more on the legal perspectives related to patients’ PGHD use in clinical settings see McGraw et al [47-49]). Regardless, this study suggests the efficacy of collecting and using PGHD data in health care [50,51]. More research is needed to establish the effectiveness of using PGHD data in clinical care [41], to determine the best strategies for implementing these data into clinical care process, and to consider the ethical implications of these different strategies.

### Limitations

This study has several important limitations. First, Institutional Review Board restrictions limited our ability to interview patients to determine their experiences when sharing PGHD with health care professionals. Thus, we can only portray how health care professionals and study team members experienced collection, sharing, and/or use of PGHD. More research is needed to appreciate patients’ experiences. Second, to accommodate the busy schedules of health care professionals and PHD study team members, we had to conduct 3 interviews by telephone. While we recognize that bodily-based nonverbal behavioral communicates important information in an interview, we thought it was more important to get the interview by telephone, than to other wise miss interviewing someone because face-to-face was a requirement. We compared telephone interviews with other interviews (in persons and through virtual platforms), and they were not remarkably different. Third, while the validity and reliability of other types of patient experience data has been established (eg, PRO), little is known about the validity, reliability, and effectiveness of using PGHD on clinical outcomes, which were all outside the scope of our study. Fourth, the 5 studies we examined, represented a broad range of approaches for collecting and using PGHD data, which added to the richness and breadth of our findings. However, the studies themselves were small and of limited duration and scope. Thus, the study teams and health care professionals we interviewed (and their patients), did not have long-term experiences with collecting and using PGHD, and therefore, we know little about how a longer duration of PGHD collection might affect perceptions and experiences.

### Conclusions

PGHD can provide value in the outpatient setting but must be implemented with attention to patient privacy and clinic workflows. More research is needed to understand patients’ and clinicians’ long-term experiences with using PGHD [52] to distill the benefits of using PGHD in clinical care and identify strategies for optimizing the use of these tools and to establish an evidence base supporting the use of PGHD in outpatient settings.

## Abbreviations

EHR: electronic health records
EMA: ecological momentary assessment
HIPAA: Health Insurance Portability and Accountability Act
IT: information technology
ODL: Observation of daily living
PGHD: Patient-generated health data
PHD: Project HealthDesign
PRO: patient-reported outcomes
